# Automated isochronal late activation mapping for substrate characterization in patients with repaired tetralogy of Fallot

**DOI:** 10.1093/europace/euae062

**Published:** 2024-03-26

**Authors:** Eduardo Arana-Rueda, Juan Acosta, Manuel Frutos-López, Juan-Antonio Sánchez-Brotons, Carmen González de la Portilla-Concha, Pastora Gallego, Alonso Pedrote

**Affiliations:** Arrhythmia Unit, Department of Cardiology, Hospital Universitario Virgen del Rocío, Avda. Manuel Siurot, s/n, Sevilla 41013, Spain; Instituto de Biomedicina de Sevilla (IBiS), C Antonio Maura Montaner, Sevilla 41013, Spain; European Reference Network for Rare, Low Prevalence and Complex Diseases of the Heart (ERN GUARD-Heart), Hospital Universitario Virgen del Rocío, Avda Manuel Siurot s/n, Sevilla 41013, Spain; Arrhythmia Unit, Department of Cardiology, Hospital Universitario Virgen del Rocío, Avda. Manuel Siurot, s/n, Sevilla 41013, Spain; European Reference Network for Rare, Low Prevalence and Complex Diseases of the Heart (ERN GUARD-Heart), Hospital Universitario Virgen del Rocío, Avda Manuel Siurot s/n, Sevilla 41013, Spain; Arrhythmia Unit, Department of Cardiology, Hospital Universitario Virgen del Rocío, Avda. Manuel Siurot, s/n, Sevilla 41013, Spain; European Reference Network for Rare, Low Prevalence and Complex Diseases of the Heart (ERN GUARD-Heart), Hospital Universitario Virgen del Rocío, Avda Manuel Siurot s/n, Sevilla 41013, Spain; Arrhythmia Unit, Department of Cardiology, Hospital Universitario Virgen del Rocío, Avda. Manuel Siurot, s/n, Sevilla 41013, Spain; European Reference Network for Rare, Low Prevalence and Complex Diseases of the Heart (ERN GUARD-Heart), Hospital Universitario Virgen del Rocío, Avda Manuel Siurot s/n, Sevilla 41013, Spain; Arrhythmia Unit, Department of Cardiology, Hospital Universitario Virgen del Rocío, Avda. Manuel Siurot, s/n, Sevilla 41013, Spain; European Reference Network for Rare, Low Prevalence and Complex Diseases of the Heart (ERN GUARD-Heart), Hospital Universitario Virgen del Rocío, Avda Manuel Siurot s/n, Sevilla 41013, Spain; Instituto de Biomedicina de Sevilla (IBiS), C Antonio Maura Montaner, Sevilla 41013, Spain; European Reference Network for Rare, Low Prevalence and Complex Diseases of the Heart (ERN GUARD-Heart), Hospital Universitario Virgen del Rocío, Avda Manuel Siurot s/n, Sevilla 41013, Spain; Adult Congenital Heart Disease Unit, Department of Cardiology, Hospital Universitario Virgen del Rocio, Sevilla, Spain; Arrhythmia Unit, Department of Cardiology, Hospital Universitario Virgen del Rocío, Avda. Manuel Siurot, s/n, Sevilla 41013, Spain; Instituto de Biomedicina de Sevilla (IBiS), C Antonio Maura Montaner, Sevilla 41013, Spain; European Reference Network for Rare, Low Prevalence and Complex Diseases of the Heart (ERN GUARD-Heart), Hospital Universitario Virgen del Rocío, Avda Manuel Siurot s/n, Sevilla 41013, Spain

**Keywords:** Congenital heart disease, Tetralogy of Fallot, Electroanatomical mapping, Catheter ablation

## Abstract

**Aims:**

Slow conduction (SC) anatomical isthmuses (AIs) are the dominant substrate for monomorphic ventricular tachycardia (VT) in patients with repaired tetralogy of Fallot (rTF). This study aimed to evaluate the utility of automated propagational analysis for the identification of SC-AI in patients with rTF.

**Methods and results:**

Consecutive rTF patients undergoing VT substrate characterization were included. Automated isochronal late activation maps (ILAM) were obtained with multielectrode HD Grid Catheter. Identified deceleration zones (DZs) were compared with both SC-AI defined by conduction velocity (CV) (<0.5 m/s) and isthmuses of induced VT for mechanistic correlation. Fourteen patients were included (age 48; p^25–75^ 35–52 years; 57% male), 2 with spontaneous VT and 12 for risk stratification. Nine VTs were inducible in seven patients. Procedure time was 140 (p^25–75^ 133–180) min and mapping time 29.5 (p^25–75^ 20–37.7) min, using a median of 2167 points. All the patients had at least one AI by substrate mapping, identifying a total of 27 (11 SC-AIs). Isochronal late activation maps detected 10 DZs mostly in the AI between ventricular septal defect and pulmonary valve (80%). Five patients had no DZs. A significant negative correlation between number of isochrones/cm and CV was observed (rho −0.87; *P* < 0.001). Deceleration zones correctly identified SC-AI (90% sensitivity; 100% specificity; 0.94 accuracy) and was related to VT inducibility (*P* = 0.006). Deceleration zones co-localized to the critical isthmus of induced VTs in 88% of cases. No complications were observed.

**Conclusion:**

Deceleration zones displayed by ILAM during sinus rhythm accurately identify SC-AIs in rTF patients allowing a safe and short-time VT substrate characterization procedure.

## Graphical Abstract

**Graphical Abstract:**
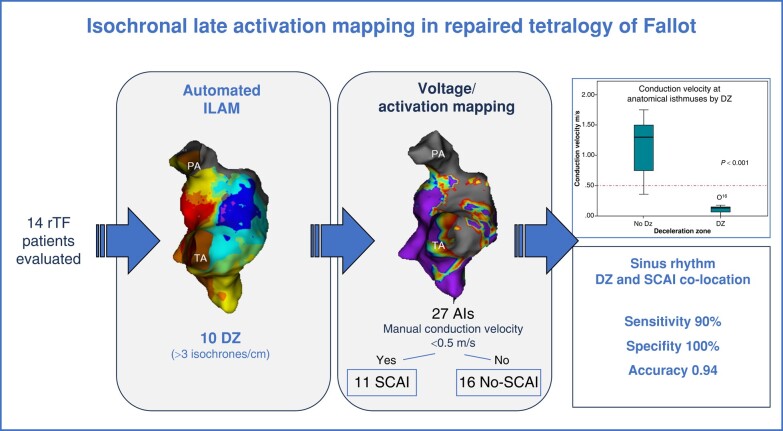

**What’s new?**

- Automated functional mapping based on isochronal late activation mapping (ILAM) accurately identifies slow conduction anatomical isthmuses in repaired tetralogy of Fallot (rTF) patients.
- The use of HD Grid Catheter in rTF patients for high-density mapping is safe and effective, allowing short mapping and procedure times.

## Introduction

The surgical approach for tetralogy of Fallot correction has progressively evolved to earlier and less invasive techniques with good results and reduced myocardial injury. Today, more than 90% of repaired tetralogy of Fallot (rTF) patients survive to adulthood.^1^ Despite this improvement in global survival, adults with rTF are still at risk for sudden cardiac death (SCD).^2,3^ Ventricular tachycardias (VT) late after surgical repair are the main cause of SCD and are related to the presence of anatomical isthmuses (AIs) between cardiac structures (valvular rings), prosthetic material (patches, valves), and myocardial scar (surgical or acquired) generated by the severity of the malformation and the mode and timing of surgical correction.^4–6^ Great efforts have been made to identify rTF patients at higher risk of presenting ventricular arrhythmias in order to prevent SCD.^3,7^ Thereby, although almost all rTF patients have AIs that could be potentially related to VT, only those with slow conduction (SC-AI) identified by 3D electroanatomical mapping (3D-EAM) represent the arrhythmic substrate of VT.^8^ The identification and transection of these SC-AIs has become the treatment of choice for VT prevention in rTF.^3,9^

Recently, the incorporation of multielectrode catheters in VT ablation procedures has allowed the development of high-density substrate mapping techniques based on a better discrimination of abnormal electrograms and a detailed identification of propagation patterns.^10,11^ Thus, this approach has become the preferred method for VT substrate identification without the inherent limitations of point-by-point mapping (highly influenced by bipole characteristics, orientation, and contact).^12,13^ Thereby, the analysis of propagation patterns during sinus rhythm (SR) with automated isochronal late activation mapping (ILAM) facilitates the identification of deceleration zones (DZ) related to VT circuits.^12^ Here, local activation maps are created tagging the latest part of the electrogram and divided into eight equal isochrones, so each isochrone shows a graphical representation of conduction velocity (CV). Areas of isochronal crowding represent an SC area, defining a DZ.^14^ Although this technology has been validated in many circumstances, resulting in shorter procedure times and superior arrhythmia free survival, data about its systematic use for VT substrate characterization in rTF are lacking.^2,15^

Our objective was to assess the utility and safety of high-density mapping based on automated ILAM for SC-AI identification in patients with rTF.

## Method

### Study population

All consecutive patients over 18 years of age with rTF referred for electrophysiological study in our centre from January 2020 to September 2023 were included. According to our clinical protocol, an electrophysiological study was indicated when the patient presented with previous spontaneous VT, an intermediate risk based on non-invasive risk factors for SCD, or prior to pulmonary valve replacement (PVR).^2,7,9^ The study was evaluated by the ethical committee of Hospitales Virgen Macarena/ Virgen del Rocio (Sevilla, Andalucia, Spain), that gave approval, and patients provided informed consent for the procedure.

### Programmed electrical stimulation

A radial arterial line was placed in every patient for continuous blood pressure monitoring. Studies were performed under conscious sedation in a drug-free state to assess morphology and haemodynamic tolerance of a clinical or targeted VT. Programmed electrical stimulation was performed at right ventricular (RV) apex and RV outflow tract (RVOT), with two drive trains (600–400 ms) and up to three extrastimuli decrementing to the ventricular effective refractory period or 180 ms. The protocol was repeated under isoproterenol infusion if no VT was inducible at baseline. Sustained VT was defined as lasting >30 s or causing haemodynamic compromise requiring termination.

### Electroanatomical mapping

#### Substrate mapping

**Figure 1:**
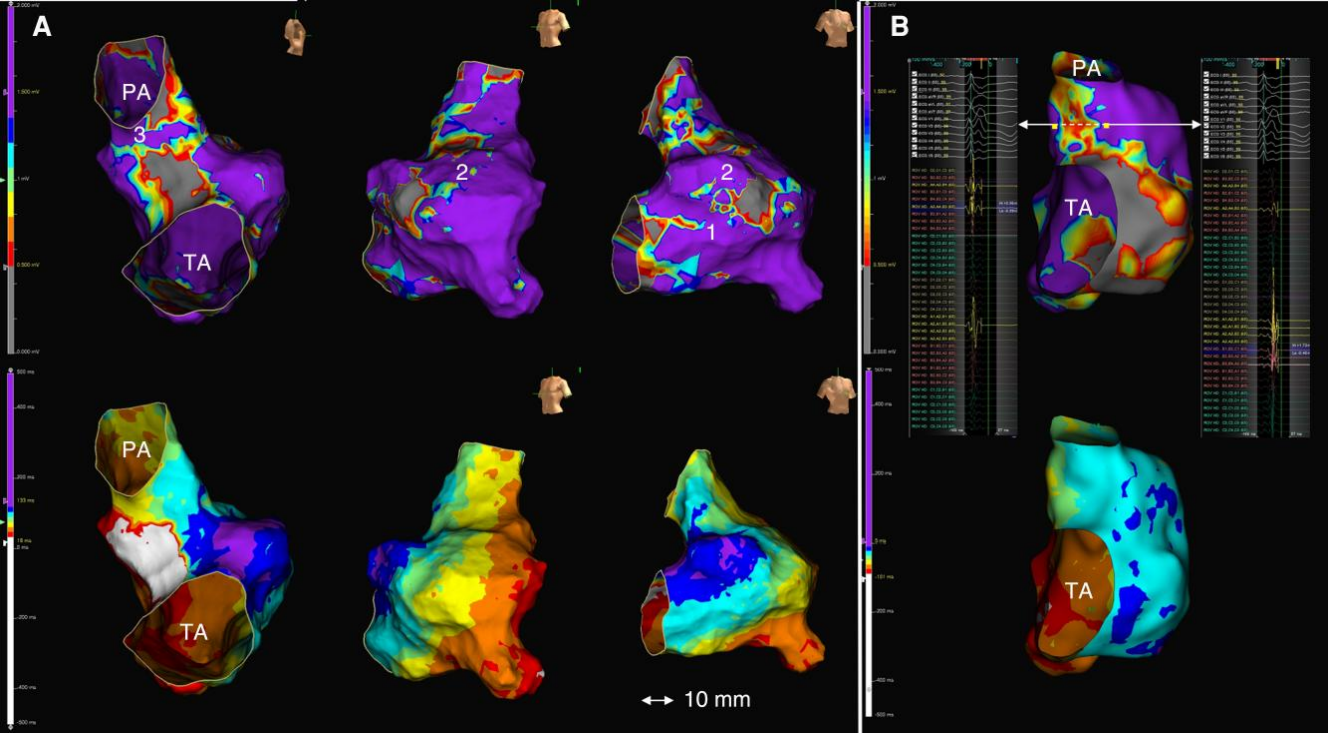
(*A*) 3D-EAM of Patient #6 showing non-contemporary rTF with RV incision. Top: substrate mapping in right lateral, LAO, and RAO views, revealing AI1, 2, and 3. Bottom: same views of ILAM showing conduction block region at VSD patch and DZ in AI3. No DZs were identified in other locations. (*B*) 3D-EAM of Patient #10 showing assessment of conduction velocity trough AI3. Top: substrate mapping delimitating AI boundaries. The length of AI was 18 mm, the conduction time was 14 ms, and the calculated CV was 1.3 m/s. Bottom: automated ILAM of RV revealing no DZ in AI3. LAO, left anterior oblique; RAO, right anterior oblique; PA, pulmonary annulus; TA, tricuspid annulus; VSD, ventricular septal defect.

A high-density 3D-EAM of the RV was performed ideally during SR with Advisor™ HD Grid Sensor Enabled™ Catheter (HDGC), using Ensite Precision/X (Abbott, Abbott Park, IL) (settings in Supplementary material online, *Figure S1*). In cases with QRS < 120 ms, mapping was performed during RVOT stimulation to avoid wave-front collision in this area. The HDGC consists of 4 splines with 4 rings of electrodes separated by 3 mm spacing, so 24 pairs of bipolar electrograms are simultaneously recorded, and the largest amplitude among all orthogonal bipole pairs is selected by the HD wave solution algorithm. A ‘paint brush’ mapping technique was applied as previously described by others.^15^ Attention was given to assess the utility and safety of HDGC in terms of accuracy, efficacy, and procedure time. A bipolar electrogram voltage <1.5 mV was considered abnormal.^8^ Areas of surgical scar or patch material were delimited by abnormal voltage amplitude and absence of local capture when pacing at high output (10 mA/2 ms). As previously described, AIs were defined as follows: between tricuspid annulus and RV incision/patch (AI1), between RV incision and pulmonary valve (AI2), between ventricular septal defect and pulmonary valve (AI3), and between ventricular septal defect and tricuspid annulus (AI4)^8^ (*Figure 1A*).

#### Functional mapping

Automated ILAM was performed using a specific software which annotates the offset of the latest component at each local electrogram (last deflection, Abbott, Abbott Park. IL), displaying eight equally distributed activation isochrones. Deceleration zones were defined as regions with >3 isochrones within a 1 cm radius (isochronal crowding), representing regions of conduction slowing. This was a more restrictive definition than the original one that identifies areas of CV < 0.6 m/s, as we wanted to identify SC-AI (i.e. CV < 0.5 m/s).^14^ Extreme conduction slowing was defined as zones with thinner isochronal crowding and continuous local fractionated activity. Line of conduction block was defined as a split potential with an isoelectric segment (>20 ms) signifying an activation gap with reversal of isochronal activation distal to the region of slowest conduction.^12^ Wave-front collision was defined by the simultaneous arrival of two opposing wave fronts occurring at the activation map. All mapping points were reviewed offline for correct annotation of the last local activation time, defined as the offset of the local bipolar electrogram deflection. Particular attention was given to isochronal distribution in pre-defined AIs, classifying them as normal-conduction AI or SC-AI after calculating the AI CV. Briefly, AI conduction time was calculated as the difference in local activation times at each side of the isthmus and the CV by dividing the distance between that points by conduction time. An SC-AI was defined as CV < 0.5 m/s^3,8^ (*Figure 1B*).

For each haemodynamically well-tolerated induced VT, the relationship between the critical part of the re-entry circuit and the identified SC-AI was determined by activation mapping, entrainment mapping (concealed fusion, post-pacing interval <30 ms of VT cycle length, stimulus-QRS equal to EGM-QRS), and/or VT termination by radiofrequency (RF). Electroanatomical isthmuses of non-tolerated VTs were identified by pacemapping [≥11/12-lead electrocardiogram (ECG) match between paced QRS and VT-QRS]. In cases with inducible VT and no identification of VT circuit in the RV, a left ventricle map was obtained.

#### Catheter ablation

The primary target for catheter ablation was the critical isthmus identified during re-entrant VT or the earliest electrogram (pre-systolic) in cases with focal pattern. Ablation was performed with a contact force open-irrigated catheter TactiCath (Abbott, Abbott Park, IL) at 40 W and temperature limit 45°C at 30 mL flow rate, aiming transection of VT-related AI by a point-by-point ablation. Radiofrequency applications were performed with contact force control. The endpoint of ablation was non-capture at high output along the ablation line (10 mA/2 ms), and isthmus conduction block was confirmed by the presence of double potentials along the line or by differential pacing. Also, post-ablation remap was systematically performed in order to verify the impact of ablation at the targeted DZ.

Ablation success for any particular VT was also defined by lack of re-inducibility.

All complications related to the procedure were recorded, including mortality and significant morbidity (vascular, valvar, or coronary artery injury, thrombus formation, heart block, pericardial effusion).

#### Follow-up

Patients were evaluated every 6 months by members of adult congenital heart disease and/or the arrhythmia departments. In patients carrying cardiac implantable electronic device, remote monitoring was provided and analysed during follow-up.

#### Statistics analysis

Categorical variables are expressed as numbers and percentages and are compared using χ^2^ or Fisher’s exact tests. Continuous data were reported as median and p^25–75^ due to the small sample size and are compared using Wilcoxon rank sum test. Linear correlation between isochrones/cm, and CV was evaluated by Spearman’s rank correlation coefficient. Measure of accuracy was performed using *F* score as class imbalance was present. To evaluate the diagnostic ability of the number of isochrones/cm for the identification of SC-AI, a receiver operating characteristics (ROC) curve analysis was performed. *P* < 0.05 was considered statistically significant. Statistical analyses were implemented using SPSS 22 (IBM Corp, Armonk, NY).

## Results

A total of 14 patients with rTF were included. Baseline characteristics are detailed in *Table 1*. Median age was 47 (p^2575^ 30–51) years and 57% were male. The median age at total repair was 4 (p^25–75^ 2.3–6.2) years. All patients had baseline SR. Median QRS width was 170 (p^25–75^ 160–192) ms, with QRS > 180 ms in six (42.8%) patients. The indication for electrophysiological study was previous spontaneous VT (14.3%), non-invasive intermediate risk for SCD (14.3%), and prior to PVR (71.4%).

**Table 1:** Patients’ baseline characteristics

	Total (14)	SC-AI (9)	No SC-AI (5)	*P*-value
Age (y)	47 (30–51)	46 (31–51)	48 (22.5–55.5)	1
Male	8 (57.1)	5 (55.5)	3 (60)	0.82
Prior shunt	2 (14.3)	1 (11)	1 (20)	0.6
Age of repair (y)	4 (2.3–6.2)	4 (2.25–6.5)	4(2–8)	1
Year of complete repair	1977 (1975–95)	1978 (1975–96)	1978 (1973–2002)	1
Transannular patch	8 (57.1)	5 (55.5)	3 (60)	0.87
Pulmonary valve replacement	1 (7)	1 (11)	0 (0)	NA
Total surgeries	1 (1–2)	1 (1–2)	1 (1–1.5)	1
Sinus rhythm	14 (100)	9 (100)	5 (100)	1
QRS duration	170 (160–192)	174 (163–197)	160 (120–180)	0.26
QRS > 180 ms	5 (35.7)	4 (44.4)	1 (20)	0.36
RBBB	13 (92.8)	9 (100)	4 (80)	0.23
RV function preserved	10 (71.4)	7 (77.7)	3 (60)	1
RV EDV (mL/m^2^)	166 (129–195)	154 (129–183)	193 (131–228)	0.6
LV function preserved	11 (78.5)	7 (77.7)	4 (80)	1
LV EDV (mL/m^2^)	72 (67–80)	71 (67–80)	75 (65–89)	1
Severe pulmonary insufficiency	10 (71.4)	6 (66.6)	4 (80)	0.25
Atrial arrhythmias	4 (28.5)	3 (33.3)	1 (20)	0.59
Study indication				
Spontaneous VT	2 (14.3)	2 (22)	0 (0)	
Pre-PVR	10 (71.4)	6 (66.6)	4 (80)	
Non-invasive intermediate risk	2 (14.3)	1 (11)	1 (20)	
Inducibility	7 (50)	7 (77)	0 (0)	0.006

Values are median and p^25–75^ or *n* (%).

EDV, end-diastolic volume; LV, left ventricular; RV, right ventricular; RBBB, right bundle branch block; SC-AI, slow conduction anatomical isthmus; VT, ventricular tachycardia.

The median duration of the procedure (skin to skin) was 140 (p^25–75^ 133–180) min, and fluoroscopy time was 9.89 (p^25–75^ 6–13) min. No complications were observed.

### Programmed ventricular stimulation

**Figure 2:**
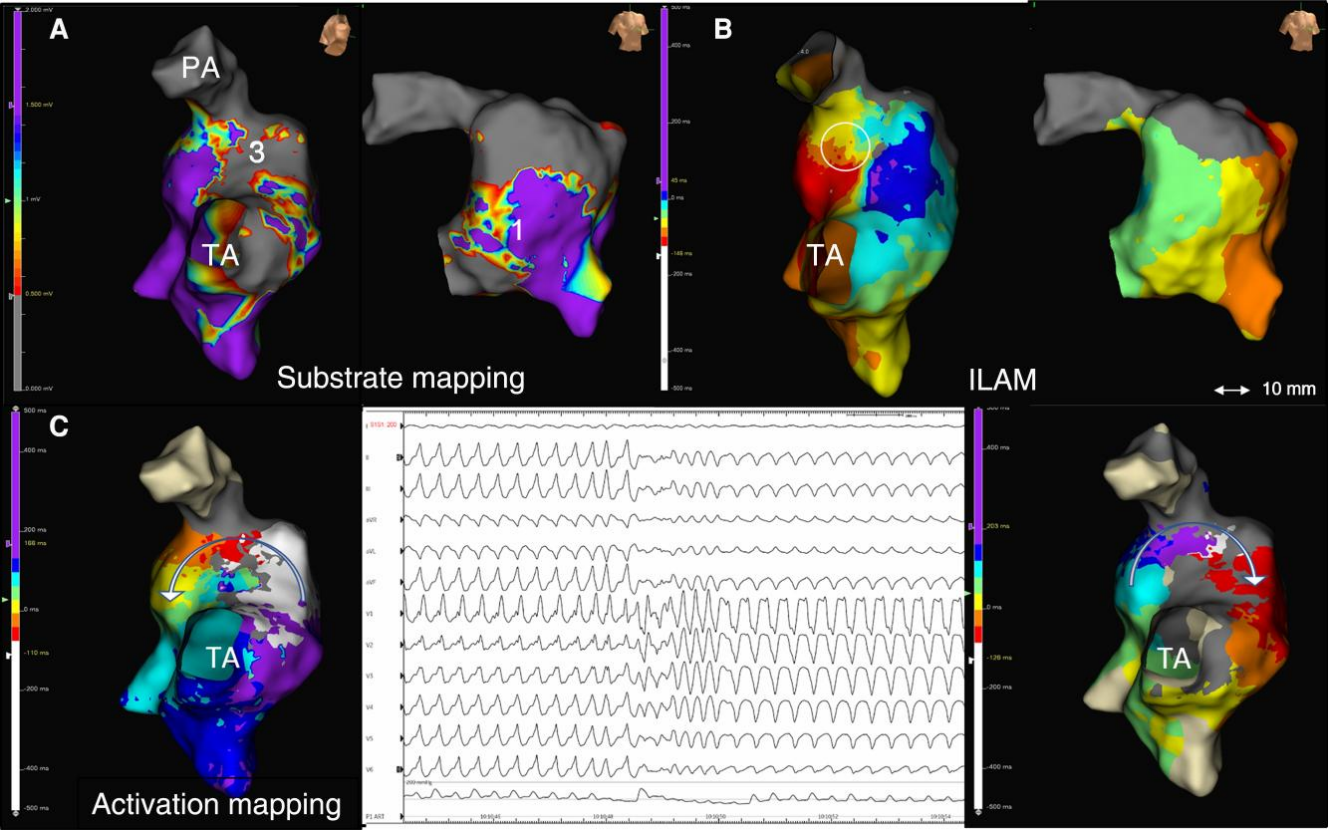
Proposed working method. 3D-EAM of Patient #7: non-contemporary rTF with large transannular patch. (*A*) Substrate mapping showing a narrow AI3 and a large AI1 (right lateral and RAO views). (*B*) ILAM reveals an isolated DZ in AI3 (circle). (*C*) A well-tolerated VT was induced that changes during activation mapping (central panel). Activation mapping of both VT morphologies was possible in a short time using HDGC, revealing the same critical isthmus at AI3 with reverse change in wave-front propagation.

Nine VTs were induced in seven patients (two patients with two different VT). The inducibility rate was 100% in patients with spontaneous VT, 50% in pre-PVR patients, and 0% in non-invasive intermediate risk patients. Four VTs showed left bundle branch block, and the remaining five VTs showed right bundle branch block morphology. An inferior axis was observed in four VTs, and five VTs had a leftward axis. Four VTs (in two patients) were well tolerated and were characterized by both activation and entrainment mapping. The remaining five VTs were hypotensive, and their exit sites were localized by pacemapping. Median cycle length of well-tolerated VTs was significantly longer than hypotensive VTs (345 p^25–75^ 333–356 vs. 240 p^25–75^ 190–255 ms; *P* = 0.029). Seven VTs were related to AI3. Patient #7 had two VTs dependent on the same AI3 (*Figure 2*), and Patient #8 showed two VTs related to left ventricular substrate. Eight VTs were macro–re-entrant, and one was considered focal or micro–re-entrant (left ventricle, Patient #8).

### High-density mapping

3D electroanatomical mapping was performed during SR with intrinsic QRS in 11 cases, whereas in 3 cases, it was obtained during RV pacing. In all cases, a HDGC was used without the utilization of sheaths. No complications due to manipulation were observed. Median high-density mapping time was 29.5 (p^25–75^ 22.6–34.7) min, acquiring a median of 19 060 (p^25–75^ 16 020–35 137) points with 2149 (p^25–75^ 1499–3386) points used. The rate of used points was 11.3%. Median total RV activation time was 151 (p^25–75^ 137–180) ms.

Substrate mapping identified 27 AIs, having all patients at least 1 AI (AI1 in 10 patients, AI2 in 3 patients, and AI3 in 14 patients). Six patients had more than one AI (three patients had AI1–3 and six patients AI1 and 3). Of the 27 detected AIs, 11 (40%) were considered SC-AI by CV calculation, SC-AI3 in 10 (71% of AI3) and SC-AI2 in 1 patient (33% of AI2). None had SC-AI1 (*Table 2*).

**Table 2:** Characteristics of the anatomical isthmuses according to the CV classification

	Total	SC-AI	Non–SC-AI	*P*-value
**Total AI**	27	11	16	
Width (mm)	14.5 (11–26.5)	11.5 (10.7–15.2)	21 (11.2–29.5)	0.22
Isochrones/cm	3 (1.5–4)	4 (4–5)	2 (1–3)	<0.001
DZ	10	10	0	<0.001
Conduction velocity m/s	0.6 (0.15–1.35)	0.15 (0.11–0.18)	1.25 (0.66–1.52)	<0.001
**AI1**	10	0	10	
AI1 width (mm)	28 (13.5–31)	NA	28 (13.5–31)	NA
Isochrones/cm	1 (1–2)	NA	1 (1–2)	NA
DZ	0	NA	0	NA
Conduction velocity m/s	1.25 (1–1.5)	NA	1.25 (1–1.5)	NA
**AI2**	3	1	2	
AI2 width (mm)	7 (6–20)	6	8–20	1
Isochrones/cm	3	5	2	0.33
DZ	1	1	0	1
Conduction velocity m/s	1.3	0.4	1.3	1
**AI3**	14	10	4	
AI3 width (mm)	11 (11–16)	11.5 (11–15.5)	14 (11–16.5)	0.6
Isochrones/cm	4 (3–4.25)	4 (4–5.5)	3 (2–3)	0.026
DZ	9	9	0	0.005
Conduction velocity	0.24 (0.12–0.7)	0.15 (0.09–0.24)	0.7 (0.63–1.2)	0.002

In general, SC-AIs are coincident with DZ, presenting an inverse relationship between isochrones/cm and CV. Slow conduction anatomical isthmuses are present in the pre-defined IAs and are correctly identified by DZ.

AI, anatomical isthmus; CV, conduction velocity; DZ, deceleration zone (>3 isochrones/cm); SC, slow conduction.

**Figure 3:**
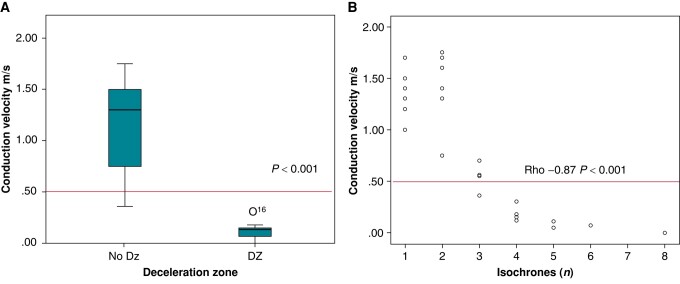
(*A*) CV at anatomical isthmuses with and without DZs. (*B*) Scatterplot showing a linear correlation between isochrones/cm and CV at AIs.

Isochronal late activation maps detected 10 DZs that co-localized with pre-defined AI. Eight patients had a single DZ (eight AI3 and one in the left aspect of infundibular septum) and one had two DZs (AI3 and AI2). Five patients had no DZs. Overall, AI3 was the most frequently associated with DZ (in 57.1% cases). Median velocity in AI with DZ was 0.15 (p^25–75^ 0.11–0.18) m/s vs. 1.25 (p^25–75^ 0.66–1.52) m/s in AI with no DZ (*P* < 0.001). A linear correlation between number of isochrones/cm and CV was present (rho −0.87; *P* < 0.001) (*Figure 3*). Deceleration zone defined by >3 isochrones/cm correctly identified SC-AI < 0.5 m/s (area under ROC curve 99%, *P* < 0.001; confidence interval 0.96–1) with 90% sensitivity, 100% specificity, and accuracy of 0.94 (see Supplementary material online, *Figure S4*). The presence of DZ was related to inducibility (*P* = 0.006) and co-localized with the critical isthmus of induced re-entrant VTs in 100% cases, 88% of total induced VTs.

### Ablation

**Figure 4:**
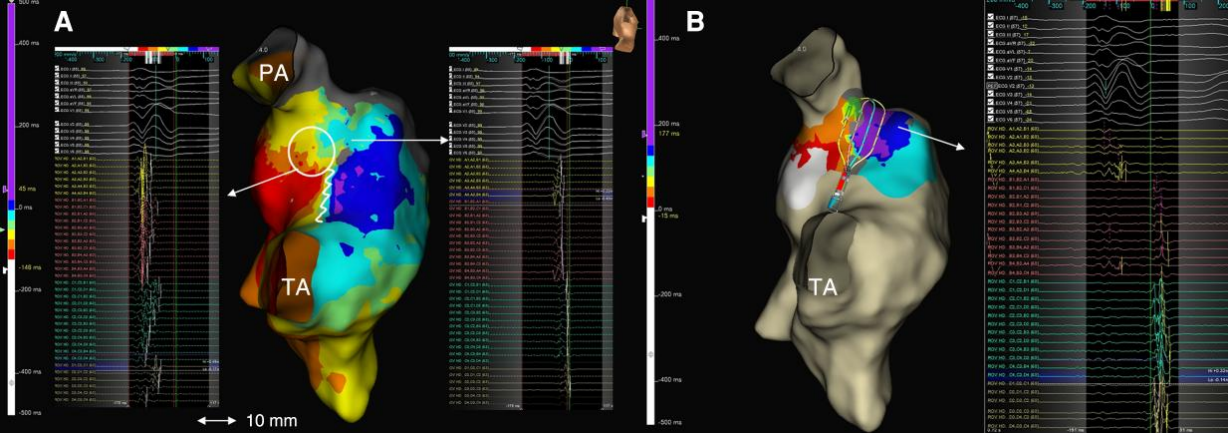
ILAM pre- and post-ablation. (*A*) DZ (circle) corresponding to a calculated local CV of 0.3 m/s in AI3; the zig-zagged line represents the block of conduction at VSD patch level. The pre-ablation analysis of the local activity showed pathological continuous activity along the AI3. (*B*) ILAM post-ablation shows a line of block with gap of isochrones and reversal of activation distal to ablation line at AI3. HDGC allows the registry of the entire area of interest, recording electrograms proximal, in the line of block with split potentials and distal simultaneously.

Catheter ablation was performed in seven (50%) patients, all induced and with SC-AIs identified in SR. The remaining seven patients were not ablated based on a combination of factors: gave no consent to RF ablation (1; 7%), were not inducible (7; 50%), and/or had no SC-AI (5; 35.7%). AI3 was targeted in six patients, and in one patient, a retroaortic approach was performed for endocardial left ventricular ablation (left aspect of infundibular septum and posterobasal region). Median ablation time was 5.9 (p^25–75^ 2.35–9.41) min. Conduction block of the targeted DZ was achieved in all cases. Remap after ablation showed a change in the propagation pattern in all cases with reversal activation distal to ablation line (*Figure 4*). The ablation of a focal VT in posterobasal LV in Patient #8 was validated by non-inducibility. Ventricular tachycardia induction was not observed after ablation in any case. Two patients underwent ICD implantation despite effective ablation (one in secondary prevention and the other in primary prevention because of the presence of several risk factors for SCD). During a mean follow-up of 15 months (range 3–42 months), all patients remained free of documented VT episodes.

## Discussion

The main findings of this study are as follows:

1. Isochronal late activation maps allow accurate identification of SC-AI in patients with rTF. This is the first study that correlates DZ identified by functional mapping with SC-AI in this context.
2. The use of HDGC in rTF patients for high-density mapping is safe and effective, allowing short mapping and procedure times.

### Ventricular tachycardia substrate identification in repaired tetralogy of Fallot and functional mapping

Since the demonstration by Zeppenfeld and coworkers^16^ that pre-defined AIs related to surgery/malformation are the critical parts of macro–re-entrant circuits in rTF, their identification and conduction block has been shown to be the treatment of choice for the prevention of VT in rTF.^2^ Due to poor haemodynamic tolerance of most induced VTs (55% in our series), a strategy based on 3D-EAM substrate evaluation/ablation during SR has become the preferred approach. Thereby, Kapel *et al.*^8^ established a threshold of <0.5 m/s for the identification of SC-AI related to VT circuits with a 93% sensitivity and 100% specificity, becoming the gold standard for SC-AI identification. However, 3D-EAMs in these studies were based on manual annotations using a single ablation catheter, thus with the inherent limitations of bipolar mapping. The incorporation of multipolar catheters has shifted the methodology of substrate evaluation to functional mapping, allowing to analyse propagation patterns within the low-voltage areas and their response to different pacing manoeuvres.^17,18^ Thereby, regions of conduction slowing with isochronal crowding that propagates into the latest zone of activation can be identified by functional mapping displays and targeted in order to eliminate VT circuits.^14^ Aronis *et al.*^19^ demonstrated that endocardial CV is inversely related to fibrosis density measured by cardiac magnetic resonance in ischaemic patients and that areas with the slowest velocity co-localize with sites of successful VT ablation. Previous studies have shown that DZs (≥3 isochrones/cm) correlate with regions of conduction slowing below the limit considered normal (<0.6 m/s).^8,14,18^ In our series, we demonstrate that the number of isochrones/cm is inversely associated with CV with a strong negative correlation (rho −0.87, *P* < 0.001) and that a more restrictive cut-off point of >3 isochrones/cm is needed for identifying SC-AI (velocities < 0.5 m/s) with high sensitivity and specificity. Therefore, we demonstrate that rTF is an ideal substrate for automated functional mapping based on ILAM, as it clearly displays the AIs of potential interest, with adequate correlation between DZs and VT isthmuses. Also, the use of ILAM provides other advantages allowing the identification of critical areas in atypical locations, as in the left ventricular aspect of AI3 (see Supplementary material online, *Figure S2*).

### High-density mapping with HD Grid Catheter

Limited information is available on mapping and ablation times of VTs in rTF, but it is generally known that these are complex procedures that require repeated programmed ventricular stimulation and activation mapping for validation of inducibility and ablation lines, with series reporting mean procedure time of 390 min.^20^ The use of HDGC has been previously evaluated in miscellaneous paediatric and congenital heart disease populations, resulting in a high-density mapping with lower procedure time and without complications associated with its use.^15^ Our series shows a population of patients with rTF evaluated both in secondary and primary prevention for VT and represents the largest study using HDGC in this scenario. In this population, high-density maps were obtained in adequate mapping and revision times (mean 29.5 min) without complications. With this in mind, given that most of the procedure time is spent in the programmed stimulation protocol before and after ablation (which can last more than 60 min), the reduction of the mapping time while maintaining high definition is of potential interest. Finally, due to rapid acquisition of points with the 16-pole configuration, a fast and high-resolution activation map could be obtained in cases with more than one VT induction (*Figure 2*). Furthermore, physiologic changes in the activation of AIs can be demonstrated by remapping with an isochronal display after ablation, which may serve as validation of conduction block (*Figure 4*) (see Supplementary material online, *Figure S3*).

### Pre-pulmonary valve replacement evaluation and ablation

Our evaluation protocol for rTF patients systematically assesses the risk for VAs before PVR as it is common practice in most specialized centres. The rationale for such approach is the presence of risk factors for the development of VTs in the rTF population programmed to PVR and the fact that the pulmonary prosthesis may cover parts of the infundibular septum and could interfere with later attempts to transect the isthmus by RF ablation.^3,21^

In our study, the proportion of inducibility (50% of all patients, 41% in primary prevention) was similar to previous cohorts evaluating pre-PVR patients, estimated between 22.5% and 49%.^22,23^ This wide range of inducibility may be due to the type of population included. Thus, in our case, most of the patients had corrective surgery in the 1970s, a factor that has been associated with a higher risk of VT inducibility.^3^

On the other hand, it was confirmed that AI3 is almost always present in all patients evaluated, as evolving surgical strategies and techniques over the years do not prevent it. HD Grid Catheter allowed correct identification of abnormal electrograms and isochronal crowding in AI3 in all cases. In our series, RF ablation was successful in all the approached cases, despite targeting the infundibular septum in most of them. Previous series described a hypertrophic infundibular septum or the interposition of prosthetic material as the main causes of ablation failure, which can occur in up to 39% cases.^24^ As previously commented, this high rate of success may be related to targeting SC-AI before it becomes inaccessible by implanted prosthetic material (surgical or especially transcatheter pulmonary valves) as it happened in Case #8 who required a left side approach.

#### Limitations

We must acknowledge that this is not a large series of patients, but our results are consistent with those reported in other studies, and high-density mapping with automatic ILAM has been previously validated in other substrates.^12,17^ In addition, the uniformity and consistency of the findings, evaluating 27 AIs with AI3 as the main source for SC as previously reported,^23,25^ provide validity despite not being a large sample of patients. We cannot directly compare procedure times vs. point-by-point mapping, as there is no control group. Furthermore, these data has not been provided by other works previously published. However, due to the complexity of the procedures, with repeated high-density mapping and a complete pre- and post-ablation induction protocol, it seems appropriate to us to consider the reported procedure times as short. The strategy of targeting SC-AI prior to surgery or percutaneous PVR in patients without clinical VT (primary prevention), although may have many advantages, has not been shown to provide long-term benefit.^3^ However, we demonstrate how preventive AI3 blockade can be performed with a high success rate and without complications prior to PVR, so we believe it should be a strategy to be considered especially in cases requiring more complex surgery/percutaneous therapy.

## Conclusions

This series reports the feasibility and efficacy of using HDGC for functional mapping for VT substrate characterization in rTF patients. As reported in other VT substrates, automated ILAM correctly identified critical sites for VT during SR and allowed short mapping and procedure times.

## Supplementary Material

euae062_Supplementary_Data

## Data Availability

Raw data are available upon reasonable request to the corresponding author.
