# Supplementary material for: Automated isochronal late activation mapping for substrate characterization in patients with repaired tetralogy of Fallot
Source: Europace. 2024 Mar 26;26(3):euae062. doi: 10.1093/europace/euae062 (PMC10964980; doi:10.1093/europace/euae062)
Supplement: euae062_Supplementary_Data [file euae062_supplementary_data.pdf]

SUPLEMMENTARY MATERIAL

Figure 1: ILAM and VT activation settings

ILAM settings VT activation mapping settings

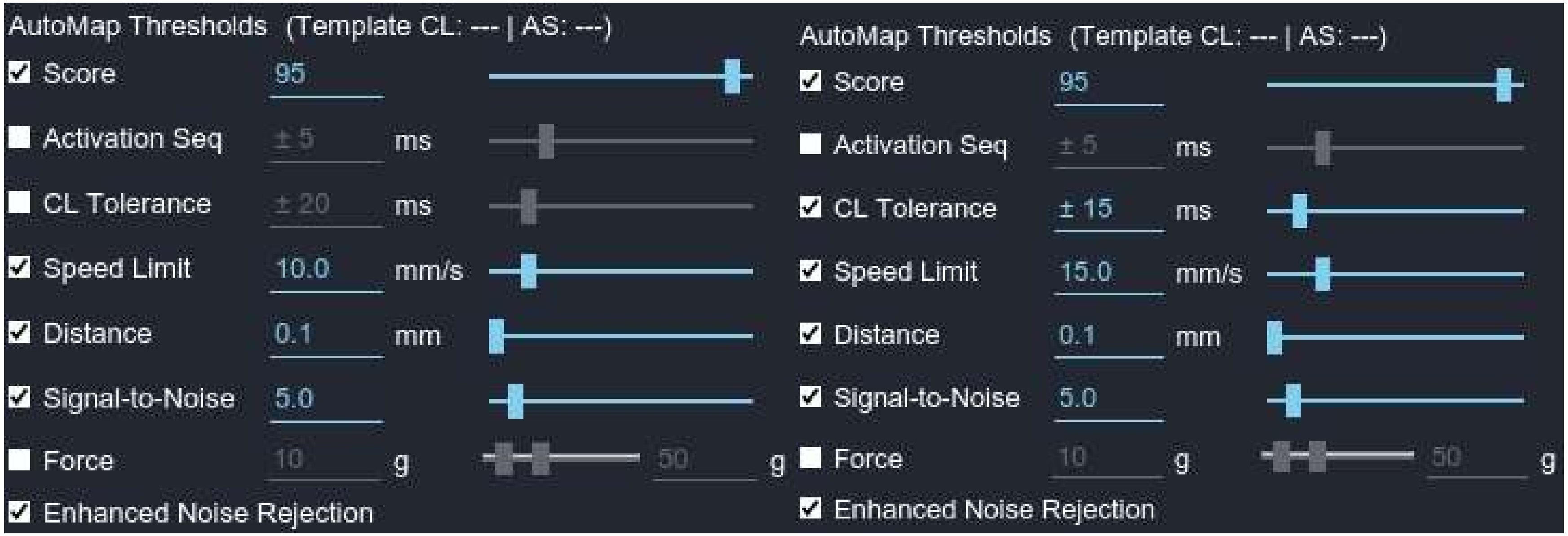

Figure 2: 3D-EAM of patient #8 with well tolerated VT. This patient had initial correction with transannular patch and VSD patching and later surgical PVR. Mapping was performed under RV stimulation A: substrate map showed a large area of pathological voltage corresponding to prosthetic material in the RVOT. (Right lateral and RAO projections). B: ILAM with craniocaudal depolarization pattern with DZ in AI3 that was not accessible due to the interposition of prosthetic material. C and D: LV mapping was performed identifying a DZ in the left aspect of infundibular septum, showing pathological electrograms. (E) VT entrainment mapping at this point showed concealed fusion and same postpacing interval than VT cycle length identifying the VT exit. Ablation in this area made VT no more inducible

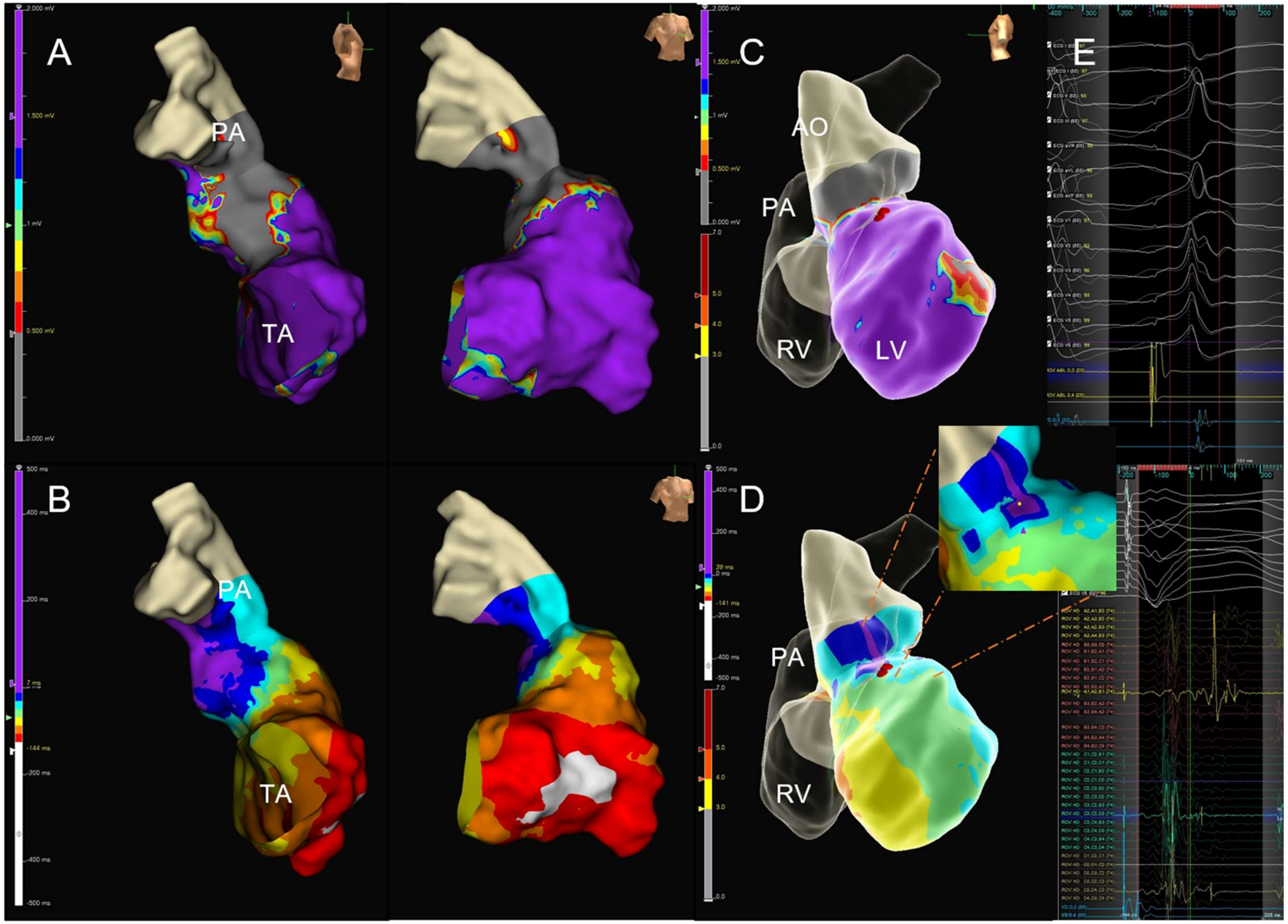

Figure 3: absence of slow-conducting isthmuses. 3D-EAM of patient #12, contemporary rTF with large transannular patch with no VT-induction during initial programmed ventricular stimulation. A: Substrate mapping showing a dilated RV with low voltage in AI3 area and a large AI1 (RAO and Right lateral views). B: ILAM revealed a baseline conduction block in AI3 area and no conduction slowing through AI1.

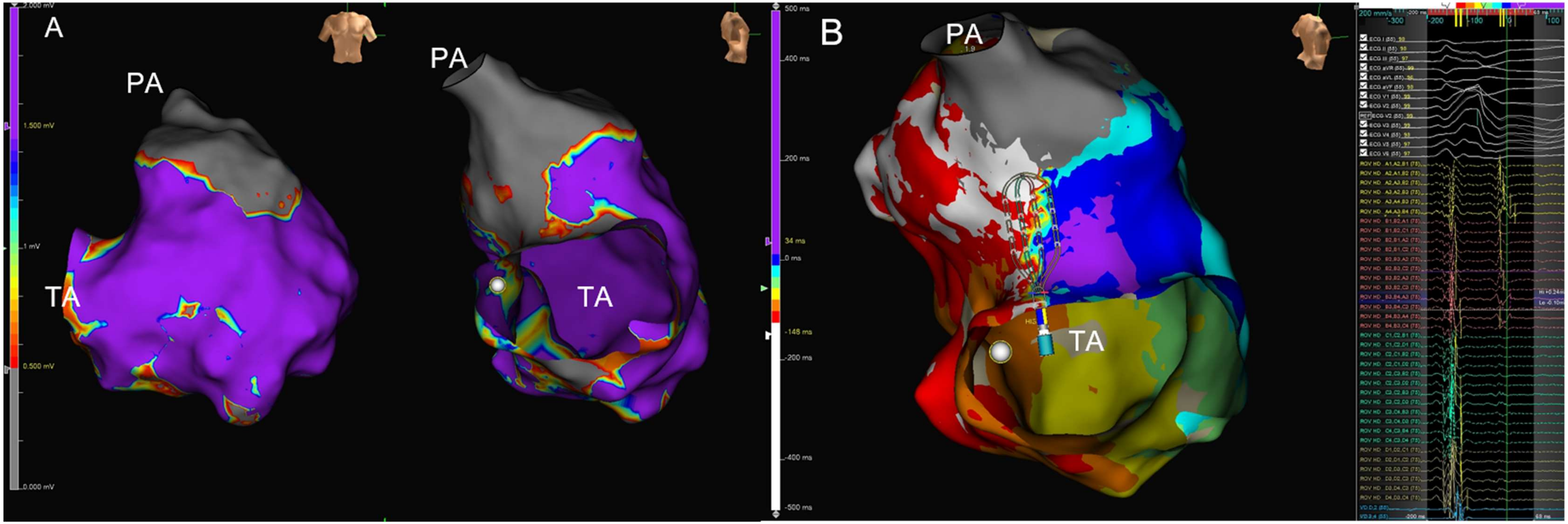

Figure 4: ROC curve of isochrones/cm for the identification of SC-AI. >3 isochrones/cm identified SC-AI with the best sensitivity and specificity. AUC: area under curve

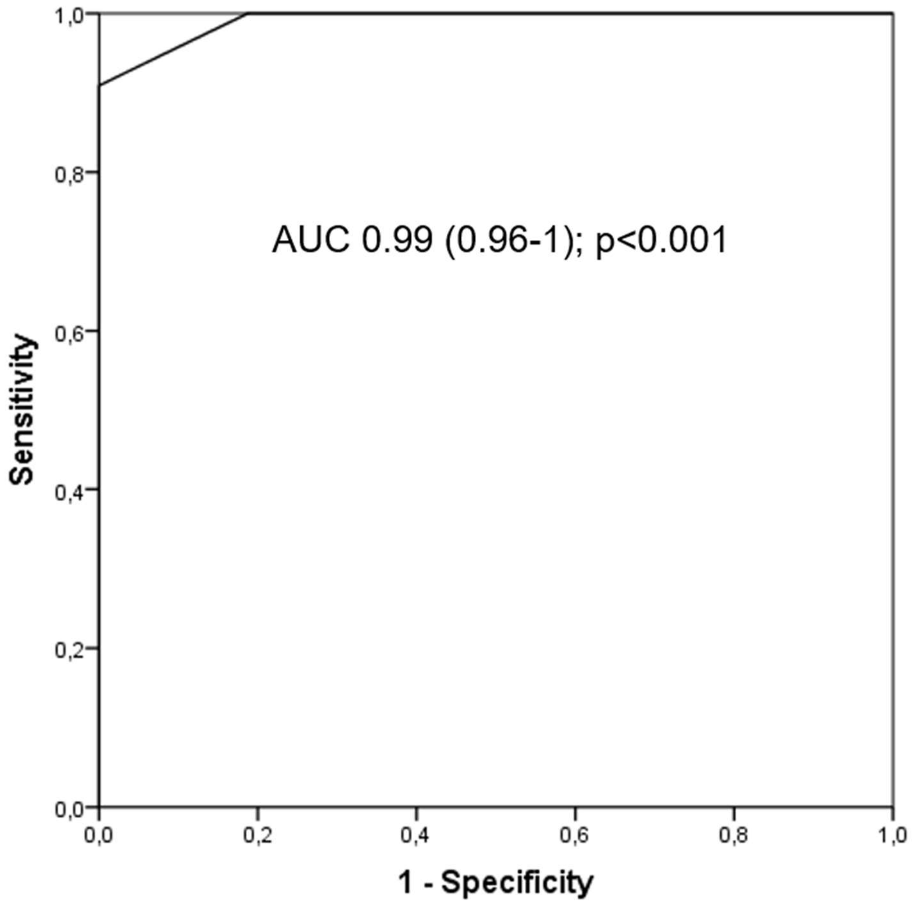

	Sensitivity	1 - Specificity
.00	1.000	1.000
1.50	1.000	.563
2.50	1.000	.188
3.50	.909	.000
4.50	.364	.000
5.50	.182	.000
7.00	.091	.000
9.00	.000	.000
